# Supplementary material for: Cyclin-dependent kinase 5 (CDK5) regulates the circadian clock
Source: eLife. 2019 Nov 5;8:e50925. doi: 10.7554/eLife.50925 (PMC6890458; doi:10.7554/eLife.50925)
Supplement: Supplementary file 2. [file elife-50925-supp2.docx]

|  |  |  |  |  |
| --- | --- | --- | --- | --- |
|  |  |  |  |  |
| Construct | NCBI | Primers | Primers sequence | Comment |
| pSCT1 |  |  |  | Langmesser et al 2008. |
| pSCT1mPer2 | NM_011066 |  |  | Langmesser et al 2008 |
| pSCT1mPer2 S-G | NM_011066 | Per2 S-G FW  Per2 S-G RW | 5’-gacagcctttcgattatggtcccattc  gcac-3’  5’gtgcggaatcgaatgggaccataatcga  gtc-3’ | Mutation of aa serine 394into glicine |
| pSCT1mPer2-V5 | NM_011066 |  |  | Schmutz et al., 2010 |
| pSCT1 ΔPasA mPer2 -V5 | NM_011066 |  |  |  |
| pSCT1 ΔPasB mPer2 -V5 | NM_011066 |  |  |  |
| Gex-4T PER2  1-576 | NM_011066 | Per2 1-576 FW  Per2 1-576 RW | 5’-ggtcgacatgaatggatacgtgga-3’  5’-gctcgagataggctagttcctc-3’ | Per2 fragment pcr product was subcloned in TOPO vector and subsequently moved into Gex-4T and inserted in the MCS using SalI/XhoI restriction sites |
| Gex-4T PER2 577-1256 | NM_011066 | Per2  577-1256 FW  Per2  577-1256 RW | 5’-ggtcgacaagaaccagcctccttg-3’  3’-gctcgagcgtctgggcctctat-3’ | Per2 fragment pcr product was subcloned in TOPO vector and subsequently moved into Gex-4T and inserted in the MCS using SalI/XhoI restriction sites |
| Gex4t Per2 1-576 S-G | NM_011066 | Per2 S-G FW  Per2 S-G RW | 5’-gacagcctttcgattatggtcccattc  gcac-3’  5’gtgcggaatcgaatgggaccataatcga  gtc-3’ | Mutation of aa serine 394 into glicine |
| Gex4t Per2 1-576 S-D | NM_011066 | Per2 S-G FW  Per2 S-G RW | 5’-gacagcctttcgattatgatccca  ttcgcac-3’  5’gtgcggaatcgaatgggatcataat  cgagtc-3’ | Mutation of aa serine 394 into aspartic acid |
| pSCT1 CDK5-HA | NM_007668 | Cdk5 (I) FW  Cdk5 (I) RW | 5’-gccaccggtatgcagaaatacgag–3’  5’-gccggatcctgggggacaga-3’ | CDK5 pcr product was subcloned in TOPO vector and subsequently moved into PSCt-1 HA and inserted in the MCS using AgeI/BamHI restriction sites |
| pSCT1 CDK5-HIS | NM_007668 | CDK5 HIS FW  CDK5 HIS RW | 5’-gggcatatgcagaaatacgacaac-3’  5’-gggatccctatgggggacagaa-3’ | CDK5 pcr product was subcloned in TOPO vector and subsequently moved into pet-15b and inserted in the MCS using NdeI/ BamHI restriction sites |
